# The S100A4 Protein Signals through the ErbB4 Receptor to Promote Neuronal Survival

**DOI:** 10.7150/thno.22274

**Published:** 2018-07-01

**Authors:** Stanislava Pankratova, Jorg Klingelhofer, Oksana Dmytriyeva, Sylwia Owczarek, Alexander Renziehausen, Nelofer Syed, Alexandra E. Porter, David T. Dexter, Darya Kiryushko

**Affiliations:** 1 Laboratory of Neural Plasticity, Department of Neuroscience, University of Copenhagen, Blegdamsvej 3, 2200 Copenhagen, Denmark; 2 Department of Pediatrics and Adolescent Medicine, Rigshospitalet, Blegdamsvej 9, 2100 Copenhagen, Denmark; 3 Research Laboratory for Stereology and Neuroscience, Bispebjerg Hospital, Bispebjerg Bakke 23, 2400 Copenhagen, Denmark; 4 John Fulcher Neuro-Oncology Laboratory, Imperial College London, Hammersmith Hospital Campus, Burlington Danes Building, 160 Du Cane Road, W12 0NN London, UK; 5 Department of Materials and London Center for Nanotechnology, Imperial College, Exhibition Road, SW72AZ London, UK; 6 Center for Neuroinflammation and Neurodegeneration, Imperial College London, Hammersmith Hospital Campus, Burlington Danes Building, 160 Du Cane Road, W12 0NN London, UK

**Keywords:** S100, S100A4, ErbB, neuroprotection, peptide

## Abstract

Understanding the mechanisms of neurodegeneration is crucial for development of therapies to treat neurological disorders. S100 proteins are extensively expressed in the injured brain but S100's role and signalling in neural cells remain elusive. We recently demonstrated that the S100A4 protein protects neurons in brain injury and designed S100A4-derived peptides mimicking its beneficial effects. Here we show that neuroprotection by S100A4 involves the growth factor family receptor ErbB4 and its ligand Neuregulin 1 (NRG), key regulators of neuronal plasticity and implicated in multiple brain pathologies. The neuroprotective effect of S100A4 depends on ErbB4 expression and the ErbB4 signalling partners ErbB2/Akt, and is reduced by functional blockade of NRG/ErbB4 in cell models of neurodegeneration. We also detect binding of S100A4 with ErbB1 (EGFR) and ErbB3. S100A4-derived peptides interact with, and signal through ErbB, are neuroprotective in primary and immortalized dopaminergic neurons, and do not affect cell proliferation/motility - features which make them promising as potential neuroprotectants. Our data suggest that the S100-ErbB axis may be an important mechanism regulating neuronal survival and plasticity.

## Introduction

Understanding the mechanisms of neurodegeneration and identification of novel pro-survival signalling cascades is crucial for development of therapies to treat neurological disorders. Neuregulin 1 (NRG) and its receptor ErbB4 are among the most important regulators of neuronal survival and plasticity in brain pathologies. The ErbB4 receptor tyrosine kinase is expressed in hippocampal and dopaminergic neurons and cerebellar granule cells, as well as in glia^1^, ^2^, and is upregulated in multiple neurological disorders^2^-^7^ with a prominent overexpression observed in neuronal populations of the affected brain areas including peri-injury regions in ischemia^4^ and brain trauma^7^, dopaminergic neurons in midbrain of Parkinson's disease patients^3^, and hippocampal neuritic plaques in Alzheimer's disease^5^. ErbB4 mainly links to the Ras-MAPK and PI3K-Akt pathways (reviewed in 1, 2). Thereby, activated ErbB4 promotes neurogenesis, neuronal differentiation and survival^2^, ^8^-^11^ exerting neuroprotective effects in models of cerebral ischemia, and Alzheimer's and Parkinson's diseases^1^, ^8^, ^12^-^15^. NRG-ErbB4 signalling also regulates synaptic function and neuronal excitability^2^, and disruption of this cascade has been shown to be a key factor in the development of epilepsy^6^, ^16^ and schizophrenia 1, 17, 18.

Recently, we have characterized a broad spectrum trophic factor in the nervous system, the S100A4 protein^19^, that could potentially affect ErbB-mediated signalling. S100A4 was initially identified as a metastasis promoter and a prognostic marker in several cancer types^20^, ^21^, but is also expressed by and secreted from non-malignant cells regulating their migration, differentiation and survival 19, 22, 23 and is markedly (over)expressed in the CNS after brain injury in rodents or humans^19^, ^24^-^26^. S100A4 belongs to a large multifunctional S100 protein family (currently 21 members) known to regulate a plethora of cell functions in tissues such as muscle, lung, bone and intestine acting both intra- and extracellularly^21^, ^27^. However, despite at least 12 members of the family being expressed in the healthy and injured nervous system, the role and signalling of S100 proteins in the brain remain poorly understood.

Likewise, signalling by extracellular S100A4 remains largely unexplored and presumably involves both a common S100 target receptor for advanced glycation end products (RAGE) and other, yet unidentified receptors^28^. Previously, we have demonstrated that extracellular S100A4 promotes neurite extension in a RAGE-independent mode^29^, ^30^, and that S100A4 also protects neurons against oxidative stress and apoptosis *in vitro* and in animal models of brain trauma and epilepsy, thus emerging as a broad spectrum neuroprotectant in the CNS^19^. These beneficial effects of S100A4 were mirrored *in vitro* and *in vivo* by two peptide mimetics of S100A4 that we designed, H3 and H6, encompassing the neurotrophic motifs of the protein. The H3-peptide shared high homology within the S100 family and the H6-peptide represented a low-homology ('unique') motif in the C-terminal of S100A4^19^, ^31^. The peptides protected neurons in cell and animal models of traumatic brain injury and excitotoxicity, as well as against genetically induced peripheral nerve degeneration^19^, ^31^. Intriguingly, neuroprotection by S100A4 was not mediated by RAGE either, and another putative target for S100A4 that we have identified, the interleukin-10 (IL-10) receptor, was only partially involved^19^, strongly suggesting the existence of additional neurotrophic pathways for S100A4. To determine these pathways, we indentified two important previous findings: (i) S100A4 activates both Ras-MAPK and PI3K-Akt cascades in neurons^19^, ^30^ and (ii) S100A4 affects EGFR (ErbB1)/ErbB2 signalling in mouse embryonic fibroblasts^32^. Based on this data, we hypothesised that the mechanism behind the neuroprotective effect of S100A4 involves ErbBs of one, or more subtypes.

Here we show that S100A4 uses the ErbB4/ErbB2 signalling axis to increase neuronal survival. We also demonstrate that S100A4 binds to the ErbB4 ligand NRG and that the S100A4/NRG interaction is important for neuroprotection by S100A4. Furthermore, peptides derived from neurotrophic sites of S100A4 protect neurons in cell models of Parkinson's disease and interact with/signal through ErbB. The peptides do not affect cell proliferation or motility making them promising candidates for development of specific neuroprotective therapies. Together, our findings for the first time link the S100 family proteins with the ErbB signalling cascade, suggesting a novel mechanism of neuroprotection in the injured brain, and introduce S100A4 peptide derivatives as neuroprotectants potentially suitable for a broad range of therapeutic applications.

## Results

### S100A4 binds to and exerts its pro-survival effect via ErbB receptors

**Fig 1:**
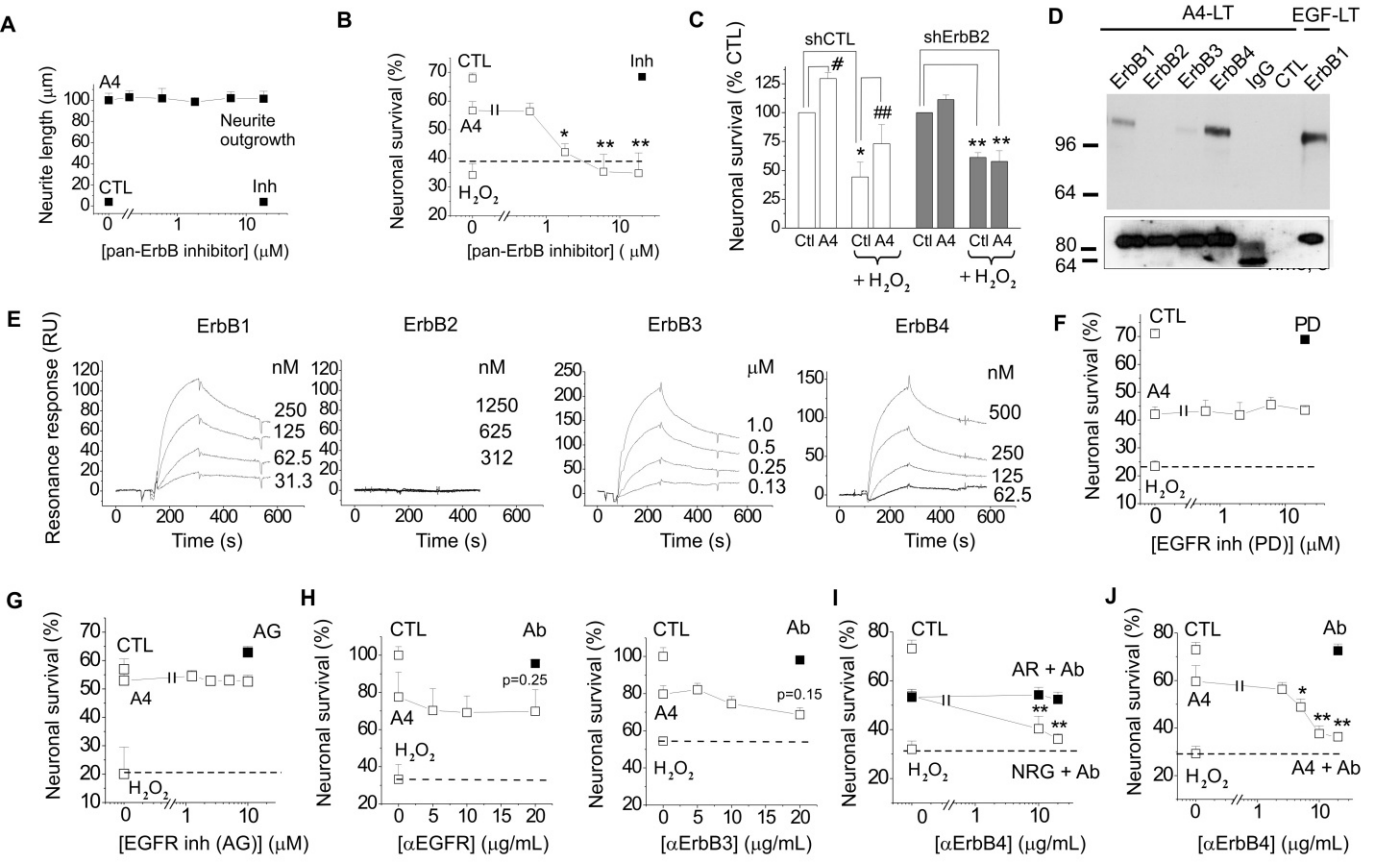
** S100A4 interacts with ErbB receptors and protects neurons *in vitro* via ErbB2 and ErbB4.** (**A, B**) Pharmacological blocker of ErbB (PD158780) does not affect the S100A4-induced neurite outgrowth (**A**), but abolishes the pro-survival effect of S100A4 in the H_2_O_2_-treated neurons (**B**). The ErbB inhibitor alone (**A**, **B**, ■) has no effect. Ctl, neurite outgrowth from untreated controls; Inh, treatment with PD158780 (20 μM) alone. In (**A**,** B**), S100A4 is used at 20 μM, four independent experiments. (**B**) CTL, neuronal viability in untreated controls, ANOVA vs cultures treated with H_2_O_2_ and S100A4 in the absence of inhibitor (A4). (**C**) Knockdown of ErbB2 reduces S100A4-induced neuroprotection (A4, 20 μM). For shRNA treatment, the survival of transfected neurons was normalized to untreated controls (100%). *, vs untreated; #, S100A4-untreated vs S100A4-treated cells, two-way ANOVA. Here and henceforth, results are expressed as means ± SEM. *p < 0.05; **p < 0.01; ***p < 0.001. (**D**) S100A4 interacts with ErbB receptors in solution. Immunoblot of the cross-linked Sulfo-SBED-S100A4 and Fc-fusion constructs of ErbB1, 2, 3, 4 or mouse immunoglobulin G (IgG) and cross-linked. Representative of two independent experiments. (ctl) Binding of sulfo-SBED-S100A4 to Protein A beads with no prey (negative control). Lower panel, loading controls. IgG, double band corresponds to partially reduced heavy and light IgG chains. (**E**) SPR binding of S100A4 to immobilized ErbB1, 2, 3 and 4, representative of two independent experiments. (**F, G**) Pharmacological blockers of EGFR PD153035 (**F**) and AG1478 (**G**) do not inhibit the pro-survival effect of S100A4 in neurons subjected to oxidative stress *in vitro*. The EGFR inhibitors alone (**F**, **G**, ■) have no effect. (**H**) Inhibitory antibodies to EGFR or ErbB3 do not have a significant effect on the S100A4-induced survival in the H_2_O_2_-treated (□) or untreated (■, Ab) neurons, four to five independent experiments. **(I)** Inhibitory antibodies to ErbB4 (Ab) do not decrease neuroprotective effect of EGFR ligand amphiregulin (AR), but specifically block neuroprotection by the ErbB4 ligand neuregulin (NRG). (**J**) Inhibitory antibodies to ErbB4 abolish the pro-survival effect of S100A4 in the H_2_O_2_-treated neurons. Antibodies alone (■, Ab) have no effect. **H-J**, CTL, neuronal viability in untreated controls, ANOVA vs cultures treated with H_2_O_2_ and S100A4 in the absence of antibodies (A4).

The involvement of ErbB receptors in S100-induced effects in neural cells has not been reported on previously; however, our earlier results indicated that S100A4 signal through ErbB1 in fibroblasts^32^. We therefore investigated whether trophic effects of S100A4 in neurons can also be mediated by ErbBs. As a test system, we used cultured hippocampal neurons, which express ErbB receptors 1, 33, 34 and in which S100A4 is known to induce neuritogenesis and increase survival during oxidative stress^19^, ^29^, ^30^. The general ErbB kinase inhibitor PD158780 did not affect the S100A4-induced neurite outgrowth (Fig 1A), but blocked the pro-survival effect of S100A4 in neurons treated with an oxidative stress inducer H_2_O_2_ (Fig 1B), indicating that the S100A4-induced neuroprotection depends on ErbBs, whereas neurite extension promoted by S100A4 is likely mediated by other mechanism(s). Since ErbB2 is only known to signal in complex with other ErbB receptors^1^, these results also suggested that S100A4 could bind ErbB1, 3, or 4 with ErbB2 possibly serving as a linker to downstream signalling cascades. Corroborating this suggestion, knockdown of ErbB2 *in vitro* abolished neuroprotection by S100A4 (Fig 1C). Interestingly, S100A4 also increased neuronal survival rates in cultures electroporated with control shRNA but not treated with H_2_O_2_ (Fig 1C) most probably reflecting the protein counteracting the decrease in neuronal viability following transfection. To further study the S100A4-ErbB interaction, we performed label transfer cross-linking experiments of S100A4 with ErbB1-4 receptors in solution (see Materials and Methods for details). We observed transfer of the biotin label to ErbB1, ErbB3 and ErbB4 (Fig 1D), indicating complex formation between S100A4 and these receptors. No S100A4 binding with ErbB2 or control antibodies (IgG) was detected (Fig 1D). Since label transfer only qualitatively detects protein interactions with the intensity of visualized bands not directly reflecting the binding affinity, we quantitatively investigated the S100A4-ErbB binding using surface plasmon resonance analysis (SPR). S100A4 directly interacted with ErbB1, ErbB3 and ErbB4 in SPR (Fig 1E, Suppl Fig 1A) binding to ErbBs immobilized on a sensor chip with the apparent affinities S100A4:ErbB1/ ErbB3/ErbB4, Kd = 21.4 ± 5.5/58.0 ± 30.0/66.0 ± 26.9 nM, calculated based on binding kinetics. No S100A4-ErbB2 binding was detected (Fig 1E). Taken together, this data indicated that S100A4 interaction with ErbBs could be the mechanism behind the pro-survival effect of S100A4 in neurons.

### S100A4-induced neuronal survival involves ErbB4 and its ligand neuregulin

**Fig 2:**
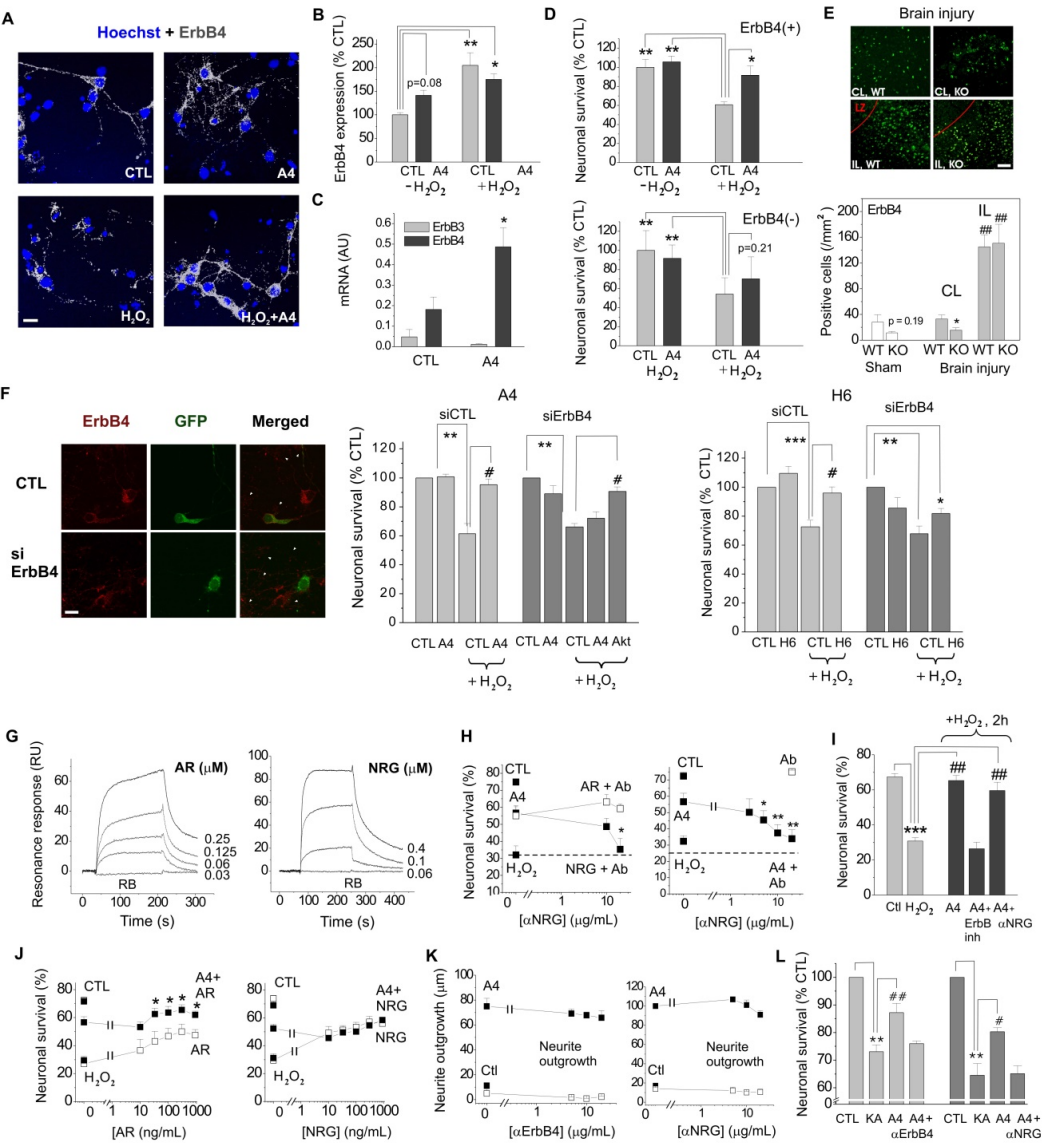
** The S100A4-induced neuroprotection involves ErbB4 and neuregulin**. **(A, B)** ErbB4 expression in hippocampal neurons is upregulated by oxidative stress (H_2_O_2_). (**A**), representative confocal images of hippocampal neurons, treated with H_2_O_2_, S100A4 or H_2_O_2_ +S100A4 (10 μM) for 24 h. Double staining ErbB4 and Hoechst to detect pyknotic nuclei. Images of ErbB4 expressing cells are binarized with a threshold of 0.25 of maximal brightness (see text for details). (**B**) ErbB4-expressing neurons are more resistant to oxidative stress compared to non ErbB4-expressing cells. Expression is normalized to untreated controls (100%), two-way ANOVA with Tukey's post-test, *, vs untreated, four independent experiments.** (C)** Treatment with S100A4 (20 μM, 24 h) upregulates the ErbB4, but not the ErbB3 mRNA in cultured hippocampal neurons, assessed by qPCR. Paired *t*-test, three to four independent experiments. (**D**) Pro-survival effect of S100A4 in neurons with moderate/high or low expression of ErbB4 (ErbB4(+) and ErbB4(-), respectively) following H_2_O_2_-induced oxidative stress. CTL, neuronal viability in controls not treated with S100A4, two-way ANOVA with Tukey's post-test, four independent experiments. (**E**) ErbB4 expression in the motor cortex (sham-operated animals) or in the sublesion zone after cryolesion ipsi- (IL) and contralateral (CL) to traumatic brain injury in WT and S100A4^-/-^ mice (KO), representative images and quantification. LZ, lesion zone. Number of animals, 8/6/4 (WT/KO/Sham). Two-way ANOVA, *, vs CL WT; #, vs Sham WT. (**F**) Left, representative confocal images of untreated (CTL) or ErbB4 siRNA-transfected (siErbB4) hippocampal neurons. White arrowheads indicate low- or non-ErbB4-expressing cells. Right, knockdown of ErbB4 reduces S100A4- and H6-induced neuroprotection (A4, H6, 20 μM). For siRNA treatment, the survival of transfected neurons was normalized to untreated controls (100%). *, vs untreated; #, S100A4-untreated vs S100A4- treated cells, two-way ANOVA. **(G)** SPR binding of amphiregulin (AR) or neuregulin (NRG) to the immobilized S100A4, representative of three independent experiments. RB, running buffer. (**H**) Inhibitory antibodies to neuregulin (Ab) specifically decrease neuroprotective effect of neuregulin (NRG) without affecting that of amphiregulin (AR) (left) and abolish neuroprotection by S100A4 in the H_2_O_2_-treated neurons (right). Antibodies alone (□) have no effect. (**I**) Neuroprotection by S100A4 (20 μM) depends on ErbB, but not on NRG at an early stage of neurotoxicity (2 h of H_2_O_2_ treatment). Assessed by Hoechst staining in living hippocampal neurons, ErbB inh (PD158780), αNRG, inhibitory antibodies to NRG. *, vs untreated; #, vs H_2_O_2_ only- treated cells, two-way ANOVA, three independent experiments. **(J)** Neuroprotection by S100A4 (A4, ■) is additive with the pro-survival effect of amphiregulin (AR, □, left), but not with that of neuregulin (NRG, □, right). (**I, J**). CTL, neuronal viability in untreated controls, one-way ANOVA vs cultures treated with H_2_O_2_ and S100A4 in the absence of antibodies (A4), four independent experiments. (**K**) Inhibitory antibodies to ErbB4 (left) or NRG (right) do not affect the S100A4-induced neurite outgrowth (■). Ctl, neurite outgrowth from untreated controls. Neither antibodies stimulated neurite extension from the control neurons when applied alone (**K**, □). One-way ANOVA vs S100A4 alone-treated cultures, four independent experiments. (**L**) Inhibitory antibodies to ErbB4 or NRG abolish the pro-survival effect of S100A4 in the KA-treated neurons. CTL, neuronal viability in untreated controls, set to 100%. *, vs CTL; #, vs KA only- treated cells, two-way ANOVA, four independent experiments.

We next set out to clarify the involvement of ErbB1 (EGFR) and ErbB4 in the S100A4-induced neuroprotection. Two selective pharmacological inhibitors of EGFR did not affect the neurotrophic effect of S100A4 in the oxidative stress model (Fig 1F, G). To inhibit the function of ErbB1, 3 and 4, we used previously described neutralizing antibodies to these receptors^35^-^37^. Neither anti-EGFR, nor anti-ErbB3 antibodies significantly inhibited the S100A4-induced neuroprotection even though weak evidence towards decreased neuronal survival was observed (p = 0.25 and p = 0.15, Fig 1H). In agreement with earlier reports, the anti-ErbB4 antibodies blocked the pro-survival effect of the ErbB4 native ligand neuregulin (NRG), but not that of the EGFR ligand amphiregulin (AR), thus specifically distinguishing between the EGFR- and ErbB4-mediated signalling pathways (Fig 1I). These antibodies did not bind to S100A4 itself, as demonstrated by SPR (Suppl Fig 1B; positive control, RAGE, a known binding partner of S100A4), but inhibited the neuroprotective effect of S100A4 without affecting the basal neuronal viability (Fig 1J), suggesting the involvement of ErbB4 in neuroprotection by S100A4. We, therefore, next investigated whether the neuroprotective effect of S100A4 correlated with the level of ErbB4 expression in hippocampal neurons. To this end, we utilized previous findings demonstrating that (i) ca 15-20% of cultured hippocampal neurons express detectable levels of ErbB4 at 10 DIV^38^ thus providing a 'natural knockout' model for this receptor, and (ii) that ErbB4 is upregulated during neurodegeneration and by oxidative stress^4^, ^7^, ^39^. In our setup, hippocampal neurons (10-12 DIV) were treated with H_2_O_2_ for 24 h in the presence or absence of S100A4 and double stained for ErbB4 and the nuclear marker Hoechst. Confocal images of ErbB4 expressing cells were binarized at an arbitrary threshold of 0.25×maximal (saturating) brightness in all image series and cells detectable at this threshold were designated as those with moderate/high ErbB4 expression (ErbB4(+)). The remaining cells were designated as those with low/absent ErbB4 expression (ErbB4(-)). Confirming previously published data, ca. 25% of untreated hippocampal neurons exhibited moderate/high ErbB4 expression (Fig 2A). In agreement with previous reports, ErbB4 expression was markedly upregulated by the oxidative stress induced by H_2_O_2_, the rate of ErbB4(+) cells increasing nearly two-fold (Fig 2B). Interestingly, we also found suggestive evidence that treatment with S100A4 alone may increase the number of ErbB4-expressing cells, which, however, did not reach statistical significance (p=0.08, Fig 2B). Accordingly, ErbB4 mRNA was upregulated by S100A4 nearly 3-fold in cultured neurons (Fig 2C, see Suppl Fig 2A for primer sequences). We have also detected the ErbB3 mRNA, albeit the level of expression was low and remained unaffected by S100A4 treatment (Fig 2C).

We subsequently compared the pro-survival effect of S100A4 in ErbB4(-) and ErbB4(+) neurons subjected to H_2_O_2_-induced oxidative stress. In ErbB4(+) cells, S100A4 was significantly neuroprotective (Fig 2D, upper panel). Cells with low expression of ErbB4 could not be efficiently protected by S100A4, though there was a slight evidence towards increased survival (p=0.21, Fig 2D, lower panel) suggesting possible involvement of other receptor (sub)types. Thus, the S100A4-ErbB4 axis could represent a 'hidden' neuroprotective mechanism activated as a result of S100A4 and ErbB4 overexpression following neuronal damage, with ErbB4 levels possibly also modulated by extracellular S100A4 released from glial cells.

We have previously shown that S100A4 is upregulated by brain trauma and that S100A4-deficient mice are more susceptible to neuronal damage following brain injury^19^. We thus examined whether expression of S100A4 and ErbB4 correlate in the normal and injured brain. ErbB4 levels in motor cortex of the S100A4^-/-^ and the wild-type (WT) mice were not significantly different (Fig 2E, Sham, p=0.19). In mice subjected to unilateral cryogenic brain injury, the number of ErbB4-positive cells was significantly lower in S100A4^-/-^ compared to WT mice on the contralateral (undamaged) side (Fig 2E, CL). Ipsilaterally, ErbB4 was strongly upregulated in both WT and S100A4^-/-^ animals, in agreement with previous reports demonstrating pronounced increase in ErbB4 levels after brain injury^3^, ^4^, ^7^ (Fig 2E, IL). Thus, ErbB4 expression was affected by brain injury and also correlated with S100A4 levels on the contralateral side of the injured brain.

To further investigate the involvement of ErbB4 in the S100A4-induced neuronal survival we knocked down expression of ErbB4 in hippocampal neurons (Fig 2F). We found that both neuroprotection induced by S100A4 and by its 'unique' C-terminal motif H6 were decreased by ErbB4 silencing (Fig 2F, see Suppl Fig 1B for peptide sequences). In S100A4-treated cells, this inhibition was rescued by the activator of Akt, a downstream kinase of the ErbB receptor signalling pathway (Fig 2F), thus corroborating the role of ErbB4 in the pro-survival effect of S100A4.

### S100A4-ErbB4/Neuregulin signalling confers neuroprotection in models of neuronal injury

Neuregulins (NRGs) are major ligands of ErbB receptors. Immature NRGs are transmembrane proteins, which upon proteolysis release soluble N-terminal fragments interacting with and activating ErbBs and launching signalling cascades to regulate neural development, plasticity and survival^40^. Neuregulin-1, in particular, has been associated with neuronal survival in several brain pathologies including AD, PD and ischemia^8^, ^13^-^15^, ^41^. Since we have previously shown that S100A4 interacts with the ErbB1 (EGFR) ligand amphiregulin (AR)^32^ but the S100A4-NRG interaction under native conditions has not been investigated, we next tested the specific hypothesis whether S100A4 could also bind NRG. Confirming previously published data, S100A4 immobilized on a sensor chip interacted with soluble AR in the SPR assay with the Kd = 71.3 ± 12.3 nM (Fig 2G, left). In the same experimental setup, S100A4 also bound with soluble NRG (Kd = 19.9 ± 3.9 nM, Fig 2G, right).

To confirm the role of NRG in neuroprotection by S100A4, we used inhibitory antibodies to NRG, which specifically blocked the neurotrophic effect of NRG, but not of AR, in the H_2_O_2_-treated cultures (Fig 2H, left). In the same experimental setup, anti-NRG antibodies abolished the S100A4-induced neuroprotection without affecting basal neuronal survival (Fig 2H, right). These results suggested that formation of the functional S100A4/ErbB4/NRG 'unit' might be necessary for S100A4 to protect neurons against prolonged oxidative stress (24 h in our setup). We then investigated the relative roles of ErbB4 and NRG in neuroprotection by S100A4 at the initial stage of neurotoxicity. Similar to long-term experiments, neuronal death induced by the 2 h exposure to H_2_O_2_ was strongly decreased by S100A4 treatment (Fig 2I). However, the neuroprotective effect of S100A4 was only inhibited by the pharmacological ErbB4 blocker, but not by the neutralizing anti-NRG antibodies (Fig 2I). Thus, NRG was ostensibly dispensable for the S100A4-induced pro-survival signalling during the early stage of neurotoxicity.

If the neuroprotective effect of S100A4 specifically involves its interaction with NRG/ErbB4, interference would be expected between the S100A4- and NRG-induced survival pathways, whereas the AR/EGFR-mediated survival should be independent of S100A4. Indeed, the neuroprotective effects of S100A4 and AR were clearly additive in neurons subjected to oxidative stress (Fig 2J, left). However, no additive effect was observed in cultures concomitantly treated with S100A4 and NRG, suggesting that the two proteins utilized significantly overlapping survival pathways (Fig 2J, right).

Interestingly, neither ErbB4-, nor NRG-neutralizing antibodies had any effect on the S100A4-induced neurite outgrowth (Fig 2K). Thus, the S100A4-NRG/ErbB4 axis presumably represented a 'survival-tuned' signalling mechanism, which could be common for many types of neuronal injury. Indeed, we obtained similar results in another physiologically relevant *in vitro* model of brain damage, in which neuronal death was evoked by kainic acid (KA), an excitotoxic agent inducing neuropathology characteristic for human temporal lobe epilepsy in rodent *in vivo* models^19^. Confirming our previous findings^19^, S100A4 robustly protected hippocampal neurons in this model, and this effect was abrogated by both anti-ErbB4 and anti-NRG neutralizing antibodies (Fig 2L).

### S100A4 and its peptide derivatives are neurotrophic in primary and immortalized dopaminergic neurons, the pro-survival effect involving ErbB4

**Fig 3:**
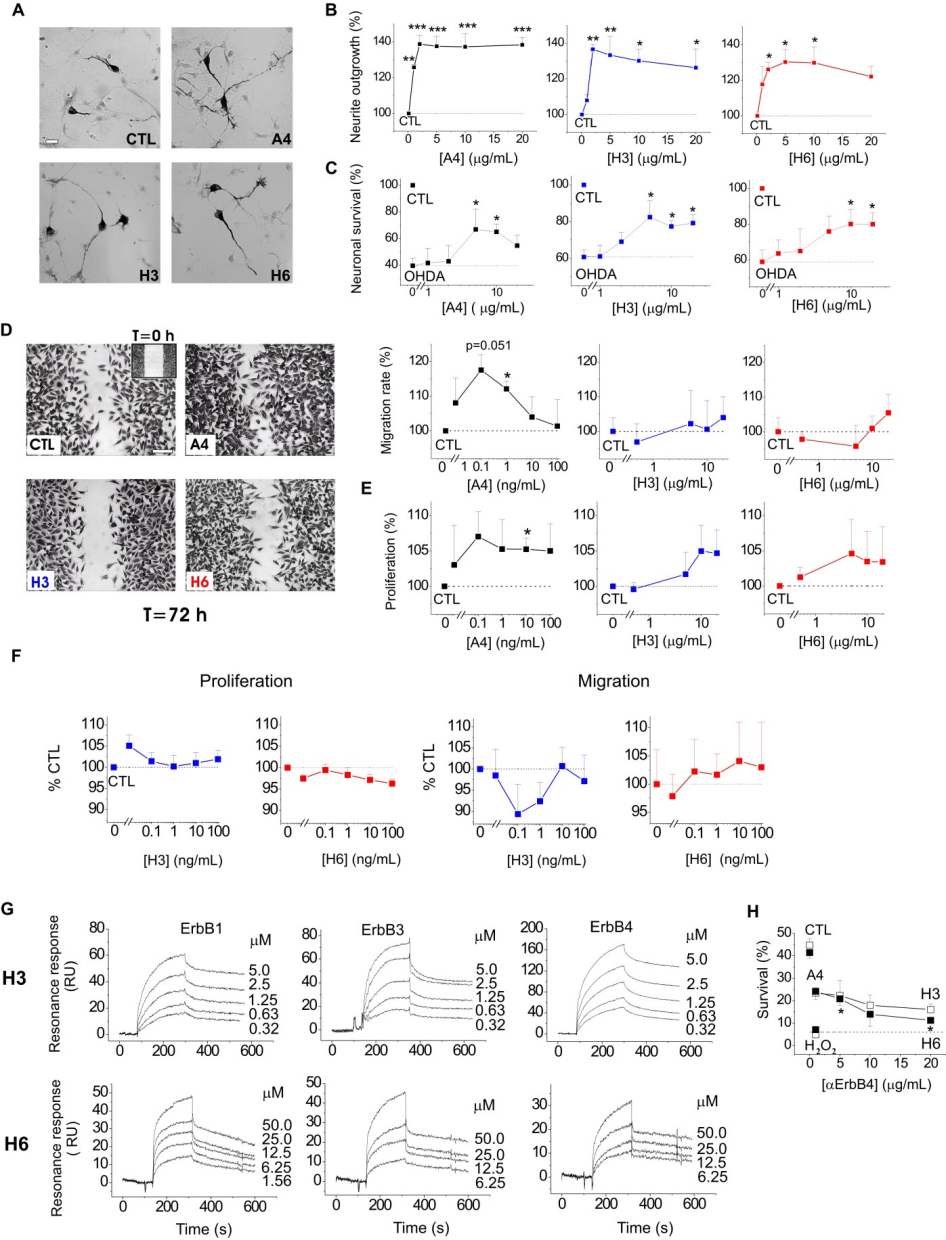
** S100A4 and its peptide derivatives act as neuritogenic and pro-survival factors in dopaminergic neurons**. **(A, B)** S100A4 (5 μM) and S100-derived peptides (H3, H6, 5 μg/ml) induce neurite outgrowth from dopaminergic neurons (representative images of TH-immunostained cultures (**A**), quantified in (**B**)). (**C**) S100A4, H3 and H6 protect dopaminergic neurons from OHDA-induced death. CTL (100%), the survival rate in the untreated controls. One-way ANOVA, * vs unstimulated cultures (**B**, neurite outgrowth) or treated with OHDA only (**C**, survival), four independent experiments.** (D**-**F)** H3 and H6 do not promote migration or proliferation of cultured human glioblastoma cells either in high (**D**, **E**) or low (**F**) concentrations. CTL (100%), the migration/proliferation rate in the untreated controls. One-way ANOVA, * vs unstimulated cultures, four to six independent experiments. (**G**) SPR binding of H3 and H6 peptides to immobilized ErbB1, ErbB3 and ErbB4, representative of two independent experiments. (**H**) Inhibitory antibody to ErbB4 partially decrease the H6-induced pro-survival effect in cultured neurons subjected to oxidative stress. CTL, neuronal viability in untreated controls, one-way ANOVA vs cultures treated with H_2_O_2_ and S100A4 in the absence of antibodies.

We next explored whether the S100A4-NRG/ ErbB4 signalling could be a mechanism behind the neuroprotective effect of the protein in other neuronal populations. It has previously been shown that S100A4 induces neurite outgrowth in dopaminergic neurons^42^ and protects them from cell death following exposure to 6-hydroxydopamine (6-OHDA), the neurotoxin which reproduces major pathological hallmarks of PD in animals, including selective dopaminergic degeneration, dopamine depletion, oxidative stress, and neurobehavioral deficits^43^. Neurotrophic peptide mimetics of S100A4, H3 and H6, have been shown to protect neurons in cell and animal models of traumatic brain injury and excitotoxicity^19^ but have not been characterized in models of PD. Supporting earlier reports, S100A4 was neuritogenic in dopaminergic neurons, and its peptide derivatives induced neurite extension comparable with that triggered by the parent protein (Fig 3A, staining for dopaminergic neuron marker tyrosine hydroxylase (TH), quantified in Fig 3B). All three compounds also had a robust pro-survival effect in the 6-OHDA neurotoxicity model in dopaminergic neurons (Fig 3C). Interestingly, the H3- and H6-peptides only mimicked the neuroprotective, but not the metastasis/ invasion-related effects of S100A4 *in vitro*. S100A4, known to increase invasiveness of astrocytic tumour cells^44^, promoted migration and, to a lesser extent, proliferation of cultured human brain glioblastomas (LN229). In contrast, the peptides had no effect on these parameters in either micromolar (Fig 3D, E) or nanomolar (Fig 3F) range of concentrations. We then investigated whether H3 and H6 interacted with and exerted their pro-survival effect via ErbBs. Interestingly, both peptides bound the immobilized ErbB1, ErbB3 and ErbB4 in the SPR experiments (Fig 3G), albeit with affinities lower than that of the parent protein (Kd values H3:ErbB1/ErbB3/ErbB4, 106.8 ± 23.1/202.8 ± 46.7/198.6 ± 53.1 nM; H6:ErbB1/ ErbB3/ErbB4, 802.2 ± 114.5/62.3 ± 18.8/217.3 ± 19.9 nM). H3 and H6 had comparable affinities to ErbB4; however, H6 had lower affinity to ErbB1 and higher affinity to ErbB3 than H3. Because both peptides interacted with ErbB4, we next examined whether their pro-survival effect in hippocampal neurons involved this receptor. The H6-induced neuroprotection was decreased by the ErbB4 neutralizing antibodies, but the H3-induced survival was not significantly affected (Fig 3H) suggesting that the H6 (C-terminal) motif of S100A4 may be important for eliciting neurotrophic responses specifically mediated by ErbB4.

**Fig 4:**
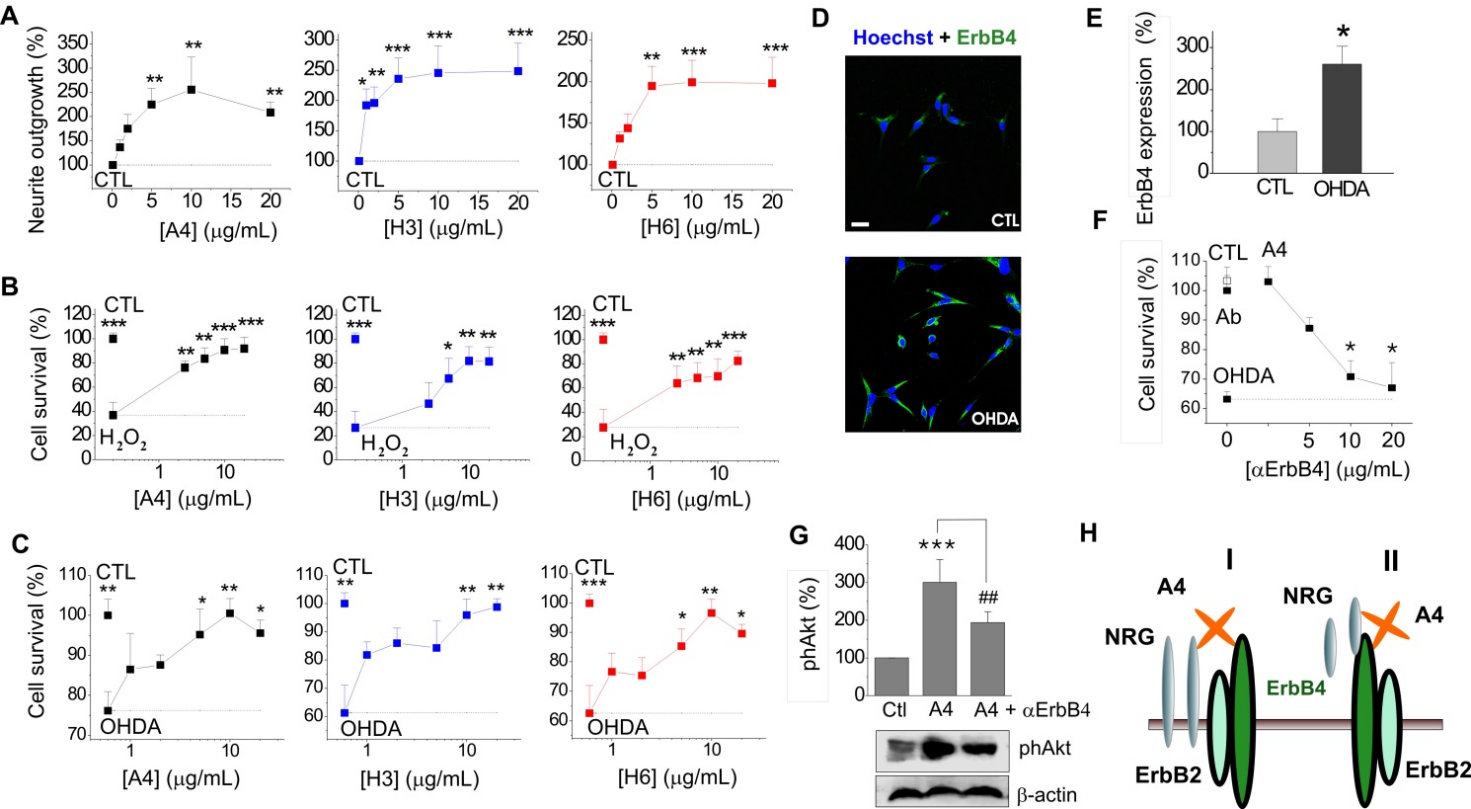
** Neuroprotective effect of S100A4 in N27 dopaminergic cells is dependent on ErbB4**. **(A-C)** S100A4 (5 μM) and S100-derived peptides (H3, H6, 5 μg/ml) promote neurite outgrowth from N27 cells (**A**) and protect N27 from H_2_O_2_-induced oxidative stress (**B**) and OHDA-induced toxicity (**C**). (**D, E**) Exposure to OHDA (100 μM, 24 h) increases the ratio of N27 cells with moderate/high expression of ErbB4 (see text for details). Representative images of cells double stained for ErbB4 and Hoechst to detect pyknotic nuclei (**D**), evaluated in (**E**), four independent experiments. (**F**) Inhibitory antibodies to ErbB4 reduce neuroprotection by S100A4 in the OHDA-treated N27 cells. Antibodies alone (■) have no effect. CTL, neuronal viability in untreated controls, one-way ANOVA vs cultures treated with H_2_O_2_ and S100A4 (A4) in the absence of antibodies. (**G**) S100A4-induced phosphorylation of Akt in N27 (A4, 10 μM) is decreased by inhibitory ErbB4 antibodies (αErbB4), five independent experiments. CTL (100%), neurite outgrowth (**A**), survival rate (**B**, **C**, **F**), ErbB4 expression (**E**), or actin-normalized phAkt levels (**G**) in the untreated controls. Two tailed *t*-test * vs CTL (**E**); ANOVA, * vs CTL (**A**, **F**), H_2_O_2_-treated (**B**) or OHDA-treated (**C**) cultures, ## vs A4 (**G**). (**H**) Signalling mechanisms involved in the S100A4-induced neuronal survival. Schematic representation of putative S100A4-NRG-ErbB functional complexes (I, II, see text for details).

To further investigate S100A4 signalling in dopaminergic cells, we employed the neuronal cell line N27 composed of immortalized TH- and dopamine transporter-positive fetal mesencephalic neurons^45^, ^46^, providing a useful *in vitro* model of dopaminergic neurodegeneration 43, 46, 47. Neurotrophic effects of S100A4 and its derivatives were fully reproduced in this system: S100A4, H3 and H6 promoted neurite outgrowth in N27 (Fig 4A) and were protective in a dose-dependent manner against cell toxicity induced by H_2_O_2_ (Fig 4B) and 6-OHDA (Fig 4C).

ErbB4 is known to be overexpressed in PD, and a similar effect is observed in toxin-based *in vitro* PD models (3 and references therein). In our experimental system, exposure to 6-OHDA resulted in more than a 2-fold increase in the number of N27 cells with moderate to high ErbB4 expression (Fig 4D, quantified in Fig 4E). Similar to the hippocampal neurons, the protective effect of S100A4 in N27 cells involved ErbB4: neutralizing antibodies to the receptor reduced the anti-apoptotic effect of S100A4 in the 6-OHDA model (Fig 4F) without affecting basal neuronal viability. The anti-ErbB4 antibodies also reduced the S100A4-induced phosphorylation of Akt, the kinase involved in canonical neurotrophic signalling pathways downstream of ErbB4^40^ and in pro-survival signalling of S100A4 in hippocampal neurons^19^ (Fig 4G). Thus, the ErbB4-PI3K-Akt cascade was at least partially responsible for trophic effects of S100A4 in N27 cells, suggesting that the S100A4-NRG/ErbB4 signalling may be involved in the S100A4-induced neuroprotection in several neuronal types.

## Discussion

In summary, we establish S100A4 as a signalling partner of ErbB4, demonstrate that the NRG/ErbB4 axis is important for pro-survival effect of S100A4 and explore biochemical and neurotrophic properties of S100A4-derived peptides. Three key aspects of our findings are to (i) provide a mechanism for the previously observed neuroprotective effect of S100A4 in the injured brain, (ii) for the first time link S100A4 with the NRG/ErbB4/ErbB2 signalling cascade in several cell models, and (iii) introduce S100A4 derivatives H3 and H6 as broad spectrum neuroprotectants potentially suitable in a range of therapeutic applications.

As we have previously shown, S100A4 robustly protects neurons in animal models of brain trauma and epilepsy, and this effect is readily reproduced in corresponding *in vitro* models, in which neuronal death is induced by oxidative stress or KA excitotoxicity^19^. Our results indicate that in both of these models, neuroprotection by S100A4 involves ErbB4/NRG signalling. Moreover, these results also provide an explanation of our previous findings showing that S100A4 activates the Ras-MAPK- and Akt- associated signalling in neurons^19^, since ErbB4 links to both of these pathways^1^, ^2^.

Interestingly, our data suggest that to exert its pro-survival effect, S100A4 interacts with and utilizes both ErbB4 and its ligand NRG. This is reminiscent of our previous findings^32^ showing that S100A4 activates EGFR and interacts with its ligand amphiregulin thereby enhancing the amphiregulin-mediated proliferation of mouse embryonic fibroblasts. However, only ErbB4, but not NRG was indispensable for neuroprotection against short-term neurotoxicity (2h oxidative stress) suggesting that whereas induction of neuroprotection by S100A4 may only require ErbB4, NRG is needed for prolonged effect of the protein. We speculate that NRG may stabilize the S100A4/ErbB4 complex and/or be recruited to ErbB4 by the S100A4 protein to act as a co-agonist (Fig 4H). Given that NRG is initially synthesized as a membrane-anchored precursor which is then shed into the extracellular space, at least two modes of S100A4/NRG/ErbB4 interaction may exist with both transmembrane (Fig 4H, I) and soluble forms of NRG (Fig 4H, II ) involved in the complex.

Although involvement of ErbB4 in pro-survival effects of S100A4 *in vivo* needs further clarification, accumulating data point to the importance of the S100A4-ErbB signalling axis in brain pathologies. There is a high similarity between the biological effects of S100A4 and the native ErbB4 ligand NRG in models of brain damage: both proteins act as antioxidants, decrease neuronal death and attenuate excitotoxicity^16^, ^19^, ^48^. We have also found that ErbB4 expression was higher in the contralateral hemisphere of S100A4 WT compared to KO animals following injury (Fig 2E). Interestingly, we have also previously detected a post-traumatic contralateral upregulation of S100A4^19^, both effects most probably following the spreading astrocytic activation, which occurs in focal brain injuries^49^, ^50^ and was also observed in our cryogenic lesion model^19^. Moreover, as it was recently demonstrated in a mouse brain injury model and human brain astrocytes, spreading astroglyosis can affect and be reciprocally regulated by the activation of ErbB receptors in reactive astrocytes^51^, ^52^. Since S100A4 can be released from stressed astrocytes^19^ and upregulate ErbB4 mRNA in cultured neurons (Fig 2C), S100A4 present in the CL hemisphere of WT animals with brain injury might directly and/or by affecting astroglyosis modulate contralateral ErbB4 expression. Taken together, these findings suggest that the two proteins may create 'hot *loci'* in the injured brain in which S100A4 released from stressed or lysed astrocytes and/or blood cells^19^ may locally bind to and activate neuronal ErbB4 receptors. Such cooperation would be facilitated by the pattern of the proteins' expression in brain pathologies, with both S100A4 and ErbB4 strongly upregulated at sites of brain injury^1^, ^3^, ^4^, ^7^, ^19^. S100A4 distribution depends on the type of brain insult, with traumatic brain injury resulting in S100A4 increase in the underlying cortex and diffuse KA-induced excitotoxicity leading to S100A4 upregulation in the hippocampus^19^. To our knowledge, no studies have to date addressed extracellular concentrations of S100A4 *in vivo*. However, our quantitative data on the S100A4 release from cultured astrocytes (≈0.02-0.2 ng/ml for 2 mm diffusion layer^19^) allows us to estimate the effective S100A4 concentration in the extracellular space (~20 nm) to be in the range of 2-20 μg/ml, which is sufficiently high for the protein to exert its biological effects. ErbB4 expression is also strongly dependent on physiological and pathological context and is likely wider than that observed *in vitro*. In particular, while in rodent hippocampal cultures ErbB4 is predominantly detected in interneurons, in the human brain, ErbB4 is also robustly expressed by hippocampal pyramidal neurons in AD, and, to a lesser extent, in age-matched controls^39^. ErbB4 is also upregulated in the midbrain of PD patients^3^ and was shown to be essential for neuroprotection in knockout mouse models of PD^15^. Activation of ErbB4 suppresses limbic epileptogenesis^16^ and protects against ischemic brain injury in animal models^41^. Thus, the S100A4-induced ErbB4 modulation may potentially play a role in many brain disorders, holding promise for future therapeutic applications. Indeed, S100A4 mimetics that we have previously developed and demonstrated to be neuroprotective in animal models of brain trauma and epilepsy, also proved efficient in two *in vitro* models of PD (Fig 3C, 4B, C). Importantly, S100A4 derivatives only mimicked neurotrophic, but not invasion-related properties of the parent protein (Fig 3D-F) thus making them suitable for further development as drug candidates. The peptides have also provided an insight in the mechanism of S100A4-ErbB interaction. In our experiments, H3 and H6 bound with ErbB4 with similar affinities; however, H6 had a significantly lower affinity to ErbB1 and higher affinity for ErbB3 than H3. Moreover, inhibition of ErbB4 diminished the H6- but not H3-induced neuroprotection (Fig 3H). Given that H3 represents a common motif in S100 proteins and H6 is a C-terminal motif unique for S100A4 these results suggests that: (i) several S100 proteins may interact with ErbB receptors and (ii) in the environment where multiple types of ErbB are available, S100A4 may have a preference for ErbB3/ErbB4 receptors, as compared to ErbB1.

Further structural studies and ErbB4 targeting strategies *in vivo* will be required to validate the S100A4/ErbB4/NRG axis in the brain. However, here, we for the first time link the S100 family protein with the ErbB signalling cascade in neurons. Given that ErbBs are involved in multiple brain functions such as neuronal differentiation, glia development, neurogenesis, and synaptic transmission, S100A4 could potentially modulate these processes. Interesting possibilities are S100A4 modulating the ErbB4-mediated synaptic plasticity, which is involved in cognition and memory formation 2, or S100A4 regulating the ErbB4-mediated neurogenesis, since both proteins are expressed at sites of neurogenesis 19, 24, 53, 54.

The focus of this study was the S100A4-ErbB4 functional link. However, our results suggest that S100A4 signalling may also involve other pro-survival receptors, in particular ErbB1 and/or ErbB3, which are similarly to ErbB4 associated with PI3K/Akt and ERK pathways and could provide a complementary pro-survival route depending on relative ErbB levels and physiological context. Indeed, neuronal ErbB expression has previously been reported in several brain regions including the developing and/or adult hippocampus^34^, ^55^-^58^. However, scarce data is available on the ErbB distribution in specific neuronal populations *in vitro* and *in vivo* depending on species, developmental stage and, importantly, the presence of neuronal injury. ErbBs are also abundantly expressed in astrocytes and can be modulated by reactive astroglyosis, thus providing an additional route for S100A4-ErbB signalling which may be particularly important in human brain where the relative astrocyte content is markedly higher than in rodents. Moreover, since a number of other S100 proteins are expressed in the nervous system and share homology with S100A4, the S100-ErbB axis may represent an important mechanism regulating neuronal survival and plasticity.

## Materials and Methods

### Peptides and recombinant proteins

Peptides (sequences H3: KELLTRELPSFLGKRT, H6: NEFFEGFPDKQPRKK) were synthesized as tetramers composed of four monomers coupled to a lysine backbone (Schafer-N, Denmark). Tetramerization was previously found to be necessary for the neuritogenic activity of S100A4^30^. Recombinant S100A4 was produced as described in 30, 59. EGF, AR, NRG1 as well as Fc-conjugated ErbB1, 2,3,4 were purchased from R&D Systems (R&D, US) as a carrier free product.

### Cell lines and animals

The rat immortalized mesencephalic dopaminergic neuronal cell line 1RB3AN_27_ (N27) was used, derived from day 12 rat fetal mesencephalic tissue and composed of immortalized cells positive for tyrosine hydroxylase^45^. N27 cells were grown in RPMI 1640 medium supplemented with 10% FBS, penicillin (100 U/mL), and streptomycin (100 μg/mL). All experiments were performed between passages 11 and 20 and at 50-80% confluence in RPMI 1640 with 1% serum.

Animals were handled in accordance with European Union legislation (European Directive 2010/63/EU). Pregnant Wistar rats (E13 or E18) were from Charles River (Denmark or UK). S100A4^-/-^ mice on an A/Sn background (8-10 wks) were generated and genotyped as described in 60, and were not phenotypically different from WT A/Sn mice, used as controls. Traumatic brain injury in mice and tissue preparation were performed as previously described^19^.

### Neuronal survival assay

Rat hippocampal (E19) and midbrain (E13) cultures were prepared according to 61 and 42, respectively. After 7 DIV, hippocampal neurons were treated with AR, NRG1 or S100A4 for 1 h, challenged with KA (300 µM) or H_2_O_2_ (60 μM, both from Sigma Aldrich, Copenhagen, Denmark), further cultured for 24 h, fixed, and stained with Hoechst 33258 (Invitrogen). Inhibitory antibodies to ErbB4 or NRG (both from Neomarkers, Fremont, CA), EGFR (D1D4J, Cell Signalling), ErbB3 (2F12, Thermo Fisher), the pan-ErbB inhibitor (PD158780, Merck, Millipore A/S, Hellerup, Denmark), or EGFR inhibitors (PD153035, AG1478, both from Sigma) were added 30 min prior to S100A4, NRG, or AR. For knockdown experiments, hippocampal neurons were prior to plating electroporated^62^ with 2 µg plasmid DNA encoding shRNA for rat ErbB2 or nontargeting shRNA (OriGene, Rockville, MD, USA), both cloned in pGFP plasmid. Alternatively, neurons (5 DIV) were transfected with 5 pMol of Stealth RNAi™ siRNA (1330001, ThermoFisher) mixed with 0.5 µg pmaxGFP-encoding plasmid (Lonza) using Lipofectamin 2000 (Invitrogen) in serum- and antibiotics-free medium according to manufacturer's protocol. After 3 DIV (shRNA) or 2 DIV (siRNA), neurons were consecutively treated with S100A4 or H6 and H_2_O_2_ as described above, further cultured for 24 h and stained with Hoechst 33258. Akt activator (SC79, Calbiochem, 4 μM) was added 1 h prior to H_2_O_2._ To confirm the knockdown of ErbB4, neurons were stained with rabbit-anti-Erb4 antibodies (Santa Cruz, sc-33040, 1:200) followed by Alexa Fluor 568-conjugated goat anti-rabbit antibody. Images were acquired using a Zeiss (Carl Zeiss, Germany) LSM 510 confocal laser-scanning microscope with an oil immersion 63×1.4NA objective (Carl Zeiss). To quantify the numbers of surviving transfected neurons, the cells were counterstained for GFP, the total number of cells did not differ between the groups. To evaluate neuronal survival in the presence and absence of ErbB4, hippocampal cultures (10 DIV) were treated with S100A4 (5 μg/ml) for 1 h followed by H_2_O_2_ (60 μM), cultured for 24 h and double stained with Hoechst 33258 and anti-ErbB4 antibodies (Ab77, Thermo-Fisher, UK). Midbrain cultures were grown for 12 days, treated with S100A4, H3 or H6 for 1 h followed by OHDA (100 μM), further cultured for 24 h and immunostained with polyclonal anti-Tyrosine Hydroxylase (TH) antibodies (PA5, 1:1000, Thermo-Fisher, UK) to identify dopaminergic neurons.

To evaluate neuronal survival, 25 (hippocampal neurons) or 50 (dopaminergic neurons) images were recorded randomly for each group in each experiment employing a Nikon Eclipse E800 microscope with a Nikon Plan ×20 objective (Nikon, Tokyo, Japan) coupled to a video camera (QImaging, Surrey, Canada). Images were acquired using the ImagePro software (Media Cybernetics, Rockville, USA). Neuronal survival was evaluated as the ratio of live (non-pyknotic) neurons to the total number of neurons (hippocampal, Hoechst staining) or as an average number of TH-positive neurons per image (dopaminergic, TH staining) using the PlabApp software (Protein Laboratory, University of Copenhagen, Denmark, 2002) as previously described 62, 63. The obtained viability levels were normalized to those in untreated controls (CTL, set to 100%).

### Neurite outgrowth assay

Freshly isolated hippocampal or midbrain neurons were plated in 8-well LabTek Permanox slides (NUNC, Denmark or UK, coated with 1 μg/ml laminin for 24 h at 37°C for midbrain cultures) at a density of 10,000 (hippocampal) or 100,000 (midbrain) cells per well, stimulated with serially diluted S100A4, H3 or H6 and grown for 24 h. Whenever applicable, pharmacological blockers and inhibitory antibodies were added 30 min prior to S100A4. After 24 h *in vitro*, hippocampal cultures were stained with Coomassie Blue R250 and midbrain cultures were immunostained for TH as described above. Neurite outgrowth was evaluated using computer-assisted microscopy as described in 61.

### ErbB4 expression studies and confocal microscopy

Hippocampal cultures were grown for 12 days, treated with H_2_O_2_ for 24 h in the presence or absence of S100A4 (10 μM) and co-stained for Hoechst to detect pyknotic nuclei and ErbB4 as described above. Images of at least 200 neurons for each group in each experiment were obtained with a Leica Laser Scanning System 2000 coupled to a Nikon Eclipse TE 200 confocal microscope equipped with an oil-immersion 60×1.4-NA objective (Nikon, Tokyo, Japan). To ensure quantitative comparability, stainings were performed concurrently for all treatments and acquisition parameters were kept constant throughout recording for each experiment. In all recorded series, background-subtracted images of ErbB4-expressing neurons were binarized at an arbitrary threshold of 0.25×maximal brightness (calculated from all images recorded in the experiment), and detected cells were designated as 'neurons with moderate to high ErbB4 expression' (ErbB4(+)). The remaining cells were designated as 'neurons with low ErbB4 expression' (ErbB4(-)).

### Sulphorhodamine B (SRB) proliferation assay

Human glioblastoma cell line LN229 was cultured in DMEM (Gibco) supplemented with 10% FBS (Gibco) in a 5% CO2 humidified incubator at 37°C. Cells were seeded in 96-well plates (Corning, New York, USA) at 2×10^3^ cells per well in DMEM supplemented with 2% FBS. Twenty-four hours post-plating LN229 cells were treated with S100A4, H3, or H6 for 72 h. Cells were then fixed with 10% tri-chloroacetate (TCA) for at least 1 h at 4°C, washed 4 times with distilled water and allowed to air dry before being stained with 0.4% w/v SRB (Sigma) for 1h. Since SRB is light sensitive, staining was done concurrently on all plates to ensure comparable results. Plates were washed with 0.1% acetic acid to remove unbound SRB and air-dried. Bound SRB was dissolved in 10 mM Tris pH 10.5 and the absorbance was measured at 490nm using an EL×800 microplate reader (BioTek, Vermont, USA). Absorbance values were normalised to untreated controls.

### Wound healing migration assay

LN229 cells were plated into wound healing inserts (Ibidi, Munich, Germany) at a density of 1.7×10^4^ cells per insert well (7.7×10^4^ cells/cm^2^) in DMEM supplemented with 10% FBS. Twenty four hours post-plating inserts were removed, wells washed with warm PBS and cells treated with H3, H6 or S100A4 in DMEM supplemented with 1% FBS for 8 h. Cells were fixed with 10% TCA for at least 1 h at 4°C and stained with SRB as described above. Images of the gap were captured using a TMS inverted phase contrast microscope (Nikon) with a DinoEye AM7023 eyepiece camera (AnMo Electronics, Taipei, Taiwan). Migration rate was evaluated as a percentage of the gap area covered by migrated cells using ImageJ software package (U. S. National Institutes of Health, Bethesda, Maryland, USA).

### Histology and morphometric analysis

Animal perfusion and brain processing were performed as previously described^64^. Immunohistochemistry was performed on 7 μm coronal sections as described in^19^, ^64^ using the anti-ErbB4 primary antibodies (1:400; Chemicon, HFR1/2G4), followed by Alexa Fluor-conjugated (Invitrogen) secondary antibodies. Negative controls were prepared identically, but the primary antibody was omitted.

Images were acquired using an Olympus BX-51 microscope and the Visiopharm Integrator System software (Visiopharm). Quantifications were performed in the sublesion area (3 sections per animal, Sham/KO/WT, n =4/6/8) using the computer-assisted Stereological Toolbox program with unbiased sampling (CAST-2, Olympus) by a researcher blind to treatment.

### qRT-PCR analysis of ErbB expression

Hippocampal neurons (3×10^6^) were seeded in 30 mm tissue culture dishes, cultured for 7 days and treated with 10 μM S100A4. Total RNA preparation, qRT-PCR and data analysis were performed as described previously^65^, primer sequences are shown in Suppl Fig 2A. All samples were run in duplicates. The relative levels of the PCR products in all samples were evaluated by the Pfaffl method using GAPDH as a house keeping gene 66.

### S100A4-ErbB binding in solution, label transfer cross-linking assay

To study S100A4-ErbB interactions, human recombinant S100A4 and EGF (positive control) were derivatized with sulfo-N-hydroxysuccinimidyl-2-(6-[biotinamido]-2-(p-azido benzamido)-hexanoamido) ethyl-1,3'-dithioproprionate (sulfo-SBED, Pierce) as specified by the manufacturer's protocol. The biotin-linker incorporation was estimated by using a Biotin Quantitation Kit (Pierce) according to supplier's protocol.

For the photochemical cross-linking Sulfo-SBED-conjugated proteins (100 nM) were incubated in dark for 30 min at RT with 50 nM EGFR-Fc, ErbB2-Fc, ErbB3, ErbB4-Fc, or as control mouse IgG (Sigma) in 100 μL TBS-Ca^2+^. To cross-link interacting proteins, the samples were irradiated with UV light (302 nm) for 5 min. Resulting complexes were precipitated with Protein A-coupled magnetic Dynabeads (Invitrogen). The beads were subsequently washed four times with 500 μL ice-cold TBS/0.1% Triton X-100. To elute precipitated proteins and to cleave the cross-linker spacer arm, the beads were resuspended in 50 μL 1x reducing sample buffer (50 mM Tris-HCl (pH 6.8), 2% SDS, 10% glycerol, 100 mM DTT and 0.1% bromphenol blue) and boiled for 5 min at 95°C. Samples were placed on magnet and supernatants were transferred to clean tubes, before loading on a 6% SDS-PAGE for Western blot analysis. Pierce High Sensitivity Streptavidin-HRP (1:10000; Thermo Scientific) was used to detect the protein bands with biotin label, and loading control bands were detected by protein A-HRP (1:16000; Thermo Scientific).

### Immunoblotting assay

N27 cells (10×10^6^ cells) were grown in low-serum (1%) culture medium for 24 h before treatment. Immunoblotting was performed as described previously 29. Rabbit anti-phospho-Akt antibodies (1:1000; Invitrogen) or rabbit anti-β-actin (1:5000; Sigma) were used. Akt phosphorylation was normalized to β-actin levels for each sample.

### SPR analysis

SPR experiments were conducted on a BIAcore 2000 system (GE-Healthcare Life Sciences, Upsala, Sweden). To study protein-protein and protein-peptide interactions, 2000 resonance units (RU) of recombinant human S100A4, the human extracellular ErbB1/Fc, ErbB3/Fc, or ErbB4/Fc chimera (R&D Systems) were immobilized covalently on a CM4 sensor chip (Biacore). Binding and data analysis were performed as described previously^29^. For each condition, two to three independent experiments were performed.

### Statistics

Statistics was performed using Origin 8 software (OriginLab) and GraphPad Prism 6 (GraphPad software, Inc., La Jolla, CA, USA) by two-tailed *t*-test, one-way ANOVA or two-way ANOVA with Tukey's, Dunnett or Sidak post-tests to identify statistically significant groups. Unless indicated otherwise, results are expressed as means ± SEM, *p < 0.05; **p < 0.01; ***p < 0.001.

## Supplementary Material

Supplementary figures.

## Abbreviations

6-OHDA: 6-hydroxydopamine
AD: Alzheimer's disease
CNS: central nervous system
ERK: extracellular signal regulated kinase
GFP: green fluorescent protein
KA: kainic acid
NRG: Neuregulin 1
PD: Parkinson's disease
RAGE: receptor for advanced glycation end products
SPR: surface plasmon resonance.
