# Supplementary material for: The S100A4 Protein Signals through the ErbB4 Receptor to Promote Neuronal Survival
Source: Theranostics. 2018 Jul 1;8(14):3977–90. doi: 10.7150/thno.22274 (PMC6071530; doi:10.7150/thno.22274)
Supplement: Supplementary file 1 — Supplementary figures. [file thnov08p3977s1.pdf]

SUPPLEMENTARY FIGURE LEGENDS

Suppl. Fig. 1. (A) SPR binding of S100A4 to immobilized ErbB1, 3 and 4. **(B)** SPR binding of ErbB4 inhibitory antibodies, 20 and 40 μg/ml (Ab 20 and 40) to the immobilized S100A4, representative of three independent experiments. RAGE (500 nM), a known binding partner of S100A4, was used as a positive control.

Suppl. Fig. 2. Sequences of primers (**A**) and S100A4-derived peptides (**B**) used in the study.

Suppl Fig. 1

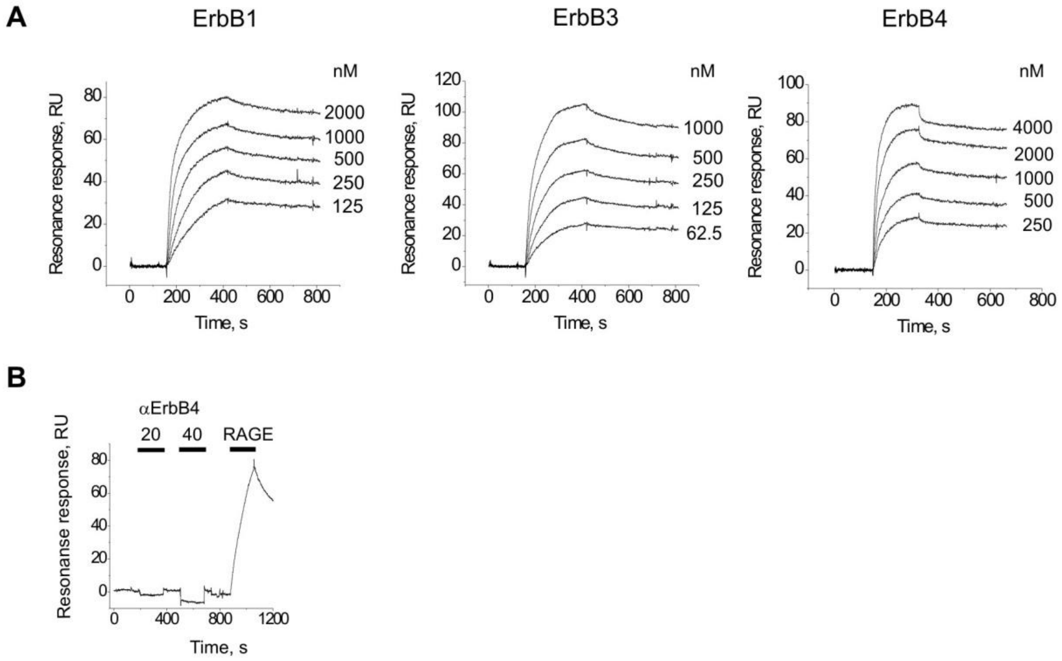
A
ErbB1
ErbB3
ErbB4
nM
Resonance response, RU
Time, s
2000
1000
500
250
125
1000
500
250
125
62.5
4000
2000
1000
500
250
B
αErbB4
20
40
RAGE
Resonanse response, RU
Time, s

Suppl Fig. 2

A

S100A4-derived peptides

H3: KELLTRELPSFLGKRT
H6: NEFFEGFPDKQPRKK

B

Sequences of primers used in the study

Gene	Forward Primer Sequence [5'3']	Forward Primer Sequence [5'-3']	Amplicon length (bp)
S100A4	GGCAAGACCCTTGGAGGAGGC	GCTGTCCAAGTTGCTCAT	193
ErbB3	TAGGTGCCAAAGGTCCAATC	TTGCTCATCAACACCTCTGC	136
ErbB4	GAACAATGTGATGGCAGGTG	TGAAGTTCATGCAGGCAAAG	115
GAPDH	AAGGTCATCCCAGAGCTGAACG	CCTGCTTCACCACCTTCTTGAT	139
